# Luminescent Nanocomposite SiO_2_/EuTTA/ZIF‑8 Loaded with Uvaol: Synthesis, Characterization, Anti-Inflammatory Effects, and Molecular Docking Analysis

**DOI:** 10.1021/acsomega.5c04682

**Published:** 2025-07-14

**Authors:** Rodrigo S. Viana, Polliane Maria Cavalcante de Araújo, Juliane Pereira da Silva, Jordana Rodrigues de Santana, Erick Gabriel Alves Ferreira, Alef Batista Bezerra Barros, Cintya D’ Angeles do Espírito Santo Barbosa, Carlos Américo Lechuga Puma, Larissa T. Jesus, Ricardo O. Freire, J. Michael Mathis, Emiliano Barreto, Severino Alves

**Affiliations:** † Technology Center, 28112 Federal University of Alagoas, Campus A.C. Simões, Tabuleiro dos Martins, Maceió, Alagoas 57072-900, Brazil; ‡ Department of Fundamental Chemistry, Federal University of Pernambuco, 50670-901, Recife, Pernambuco Brazil; § Laboratory of Cell Biology, 28112 Federal University of Alagoas, 57072-900, Maceió, Alagoas Brazil; ∥ Institute of Chemistry and Biotechnology, 28112 Federal University of Alagoas, A.C. Simões Campus, Tabuleiro dos Martins, Maceió, Alagoas 57072-970, Brazil; ⊥ Pharmacy Department, 28112 Federal University of Alagoas, Campus A.C. Simões, Tabuleiro dos Martins, Maceió, Alagoas 57072-900, Brazil; # Pople Computational Chemistry Laboratory, Department of Chemistry, 74391 Federal University of Sergipe − UFS, 49100-000 São Cristóvão, SE Brazil; 7 School of Biomedical Sciences, 12376 University of North Texas Health Science Center, Fort Worth, Texas 76107, United States

## Abstract

Asthma is a chronic inflammatory respiratory condition that requires innovative approaches for effective drug delivery. Uvaol, a natural triterpene with potent anti-inflammatory effects, holds promise for asthma treatment. However, its low bioavailability limits its therapeutic applications. To overcome this challenge, we synthesized a europium-based nanocomposite (SiO_2_/EuTTA/ZIF-8) to enhance uvaol delivery. The nanomaterials were characterized using UV–visible absorption spectroscopy, fluorescence analysis, and molecular docking simulations. Drug loading and release studies were conducted in PBS to evaluate encapsulation efficiency and controlled release properties. Cytotoxicity assays were performed to assess biocompatibility, and molecular docking was used to analyze interactions between uvaol and ZIF-8. The synthesized nanocomposite demonstrated efficient uvaol encapsulation and controlled release in PBS. Cytotoxicity assays revealed biocompatibility at low concentrations (≤10 μg/mL) and toxicity at higher concentrations (≥50 μg/mL). In addition, SiO_2_/EuTTA/ZIF-8-uvaol revealed the inhibition of the lipopolysaccharide (LPS)-induced secretion of IL-6 and TNF-α in J774 cells. Molecular docking studies highlighted hydrophobic interactions and π–π stacking between uvaol and ZIF-8, supporting stable drug-nanocarrier binding. These findings suggest that SiO_2_/EuTTA/ZIF-8-uvaol is a promising platform for improving uvaol bioavailability and enabling controlled drug delivery in asthma therapy. Additionally, europium luminescence offers the advantage of real-time monitoring, further enhancing the potential of this nanocomposite for therapeutic applications.

## Graphical abstract

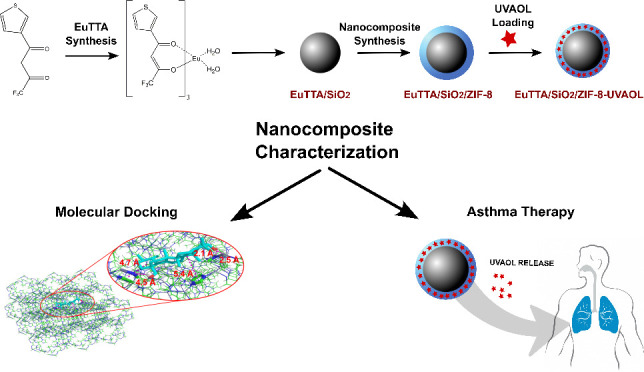

## 1 Introduction

Asthma is a chronic respiratory disease that affects 1–18% of the population worldwide.^1^ It is characterized by chronic airway inflammation, airway remodeling, bronchial hyperreactivity, and partially reversible airflow obstruction.^2^ Despite being a heterogeneous disease originating from complex interactions between genetic and environmental factors, chronic inflammation and oxidative stress in the respiratory tract remain key therapeutic targets for managing the acute exacerbations of asthma. Furthermore, several recent studies have demonstrated that epithelial-mesenchymal transition (EMT) plays a pivotal role in the pathogenesis of asthma, suggesting that the development of targeted therapies to modulate EMT can contribute to asthma treatment.^3^ While progress has been made in developing drugs for asthma and its treatment, including advancements that extend beyond traditional corticosteroid and bronchodilator therapies,^4^ challenges in treatment efficacy and accessibility persist, underscoring the need for novel therapeutic agents. Recent studies, including our own,^5,6^ have identified the natural triterpene uvaol as a promising anti-inflammatory compound. Uvaol has demonstrated significant potential in reducing inflammation in lung tissues, suggesting its utility in managing inflammatory airway diseases, including asthma.^7,8^ However, its structural properties limit its bioavailability, which poses a challenge for its clinical application. To overcome this challenge, the development of an effective drug delivery system is essential to enhance the bioavailability of uvaol and ensure its targeted delivery to lung tissues, potentially maximizing its therapeutic efficacy in asthma.

Porous nanomaterials have gained attention in recent years as effective vehicles for drug delivery, offering controlled release mechanisms that respond to physiological stimuli such as pH shifts.^9,10^ Various types of carriers, including soluble polymers, dendrimers, nanocapsules, and coordination nanostructures have been explored for this purpose. Among these, metal–organic frameworks (MOFs) have shown particular promise owing to their structural versatility and porosity. MOFs consist of metal ions coordinated to organic ligands, forming robust 2D or 3D lattice structures that facilitate controlled drug release.^11–13^ Zeolitic Imidazolate Frameworks (ZIFs), such as ZIF-8,^14^ are particularly promising MOF subtypes for biomedical applications owing to their high thermal and chemical stabilities.^15^ Additionally, the fluorescent properties of ZIF-8 offer dual functionality as a drug carrier and imaging agent, enabling the real-time tracking of drug delivery and localization in target tissues.^16–19^

Lanthanide ion (III) complexes, such as europium-based complexes, are notable for their use as biological probes because of their unique luminescent properties, photostability, extended luminescent lifetimes (>10^–4^ s), large Stokes/anti-Stokes shifts (>200 nm), and high quantum yields.^20^ Integrating europium complexes into a nanostructured carrier system containing uvaol encapsulated in a ZIF-8 matrix offers a novel strategy for managing inflammatory diseases. This approach not only improves the bioavailability of uvaol but also allows for real-time monitoring of drug distribution in lung tissues.

This study presents the synthesis, characterization, and evaluation of a Europium:ZIF-8 nanocomposite (SiO_2_/EuTTA/ZIF-8- uvaol) as an innovative material for treating inflammatory disorders with potential applications in both therapeutic efficacy and bioavailability tracking.

## 2 Materials and Methods

### 2.1 Reagents and Solutions

All experimental procedures were performed using reagents with purities greater than or equal to 95%. Succinic anhydride (99%), (3-aminopropyl)­triethoxysilane (APTES, 99%), N,N-dimethylformamide (DMF; 99%), deionized water (H_2_O), ethanol (C_2_H_6_O; 95%), zinc nitrate hexahydrate (Zn­(NO_3_)_2_·6H_2_O; 99%), 2-methylimidazole (99%), europium chloride hexahydrate (EuCl_3_·6H_2_O; 99%), 2-thenoyltrifluoroacetone (HTTA; 99%), sodium hydroxide (NaOH) aqueous solution (5% w/v), and methanol (99%) were used. The following chemicals (purchased from Sigma Chemical Co., St. Louis, MO, USA) were used: uvaol (Urs-12-ene-3,28-diol, ≥ 95% purity, PubChem CID: 92802), Dulbecco’s modified Eagle’s medium high glucose (DMEM), phosphate-buffered saline (PBS), antibiotic antimycotic solution (100×), fetal bovine serum (FBS), trypsin, Triton X-100, l-glutamine, bovine serum albumin (BSA), Tris-buffered saline (TBS), mitomycin C, and dimethyl sulfoxide (DMSO). Recombinant human TGF-β1 was purchased from PeproTech (New Jersey, USA). A stock solution of uvaol was prepared in dimethyl sulfoxide (DMSO). The DMSO concentration applied to the cell cultures never exceeded 0.1%. Neither the vehicle nor any of the compounds used at the concentrations reported in this study altered cell viability.

### 2.2 Experimental Methods

#### 2.2.1 Synthesis of SiO_2_ Nanoparticles

The SiO_2_ nanoparticles were synthesized following a modified procedure reported by Liu et al.^21^ Initially, a solution containing 0.3 mL (0.1 mmol) of succinic anhydride and 0.128 g (0.1 mmol) of (3-aminopropyl)­triethoxysilane was prepared in 50 mL of DMF under continuous stirring for 3 h at room temperature (25 °C). The resulting suspension was sonicated in an ultrasonic bath for 5 min. Subsequently, 2 mL of deionized H2O was added and the solution was stirred at the same temperature for an additional 5 h. The SiO_2_ nanoparticles were then collected by centrifugation at 13,000 xg for 10 min and washed thrice with 95% ethanol to remove any residual reactants.This surface modification introduces carboxyl (−COOH) groups onto the SiO_2_ nanoparticles, which serve as nucleation seeds during the crystallization process, promoting effective coordination with the Eu^3+^ complex, enhancing its stability, and facilitating its subsequent incorporation into the ZIF-8 matrix as a functional core in the composite structure

#### 2.2.2 Synthesis of ZIF-8 Nanoparticles

To synthesize ZIF-8 particles, 0.020 g of Zn­(NO_3_)_2_·6H_2_O was combined with 0.13 mL of deionized H2O in a vial and mixed for 5 min to form Solution A. Separately, 0.378 g of 2-methylimidazole was dissolved in 1.33 mL of H_2_O in a second vial and mixed for 5 min (Solution B). Solution A was then gradually added to Solution B with continuous stirring for an additional 5 min. The resulting product was isolated by centrifugation at 13,000 xg for 10 min and washed thrice with 95% ethanol.

#### 2.2.3 Synthesis of the EuTTA Complex

The EuTTA (Eu­(TTA)_3_ (H_2_O)_2_) complex was synthesized following the methodology of Ohlmann et al.^22^ Initially, 146 mg of EuCl·5H2O was dissolved in 2.5 mL of deionized H2O (Solution A). Separately, 0.270 g of 2-thenoyltrifluoroacetone (HTTA) was dissolved in 4 mL of ethanol and NaOH (aq) was added to this solution to adjust the pH to 6 (Solution B). Solution B was then slowly poured into Solution A followed by the addition of 40 mL of deionized H_2_O. The mixture was heated to 60 °C and stirred vigorously for 120 min. The resulting solid was collected via filtration.

#### 2.2.4 Synthesis of SiO_2_/EuTTA

To prepare SiO_2_/EuTTA, 0.366 g of EuCl3·6H2O was combined with 1.0 g of SiO2 nanoparticles in 5 mL of water (Solution A). Concurrently, 0.666 g of HTTA (3 mmol) was dissolved in 10 mL of ethanol, and the pH was adjusted to 6.0 using 5% NaOH (aq) (Solution B). Solution B was then gradually added to Solution A. After the addition of 100 mL of water, the mixture was stirred vigorously for 120 min at 60 °C. The resulting material was collected by cooling to room temperature, centrifuged at 13,000 × g for 10 min, washed multiple times with deionized water, and dried at room temperature in a desiccator.

#### 2.2.5 Synthesis of the SiO_2_/EuTTA/ZIF-8 Nanocomposite

The SiO_2_/EuTTA/ZIF-8 nanocomposite was synthesized using a two-stage process involving deposition and recrystallization. In the deposition stage, 0.1 g) was added to a Solution A containing 0.195 g of Zn­(NO_3_)_2_·6H_2_O and 1.3 mL of H_2_O, which and sonicated for 5 min (Solution A). Separately, a solution of 3.78 g of 2-methylimidazole in 13.3 mL of H_2_O was prepared and sonicated for 5 min (Solution B). Solution B was slowly added to Solution A stirred for 5 min at room temperature. The material was then collected by centrifugation at 13,000 × g for 10 min and washed thrice with deionized H2O. The intermediate product obtained at this stage was labeled SiO_2_/EuTTA/ZIF-8*. In the recrystallization stage, 0.470 g of Zn­(NO_3_)_2_·6H_2_O was dissolved in 20 mL of methanol/H_2_O Solution A (1:1) (Solution A). Separately, 0.140 g of SiO_2_/EuTTA/ZIF-8*, 1.00 g of 2-methylimidazole, and 10 mL of methanol were sonicated for 5 min (Solution B). Solution A was then slowly added to Solution B and stirred at room temperature for 5 min. Finally, the SiO_2_/EuTTA/ZIF-8 product was collected by centrifugation at 13,000 × g for 10 min, washed thrice with deionized H2O, and dried.

### 2.3 Characterization

The infrared absorption spectra were recorded at room temperature using a PerkinElmer Spectrum 400 spectrometer, covering the range from 4000 cm^–1^ to 650 cm^–1^, equipped with a diamond/ZnSe ATR cell. Thermogravimetric analysis (TGA) was performed using a Shimadzu DTG-60 thermal analyzer under a compressed air atmosphere at a flow rate of 50 mL/min. Scanning electron microscopy (SEM) images were obtained using a TESCAN Mira3 FEGSEM instrument at an acceleration voltage of 10 kV. Powder X-ray diffraction (XRD) data were obtained at room temperature using a Bruker Model D8 diffractometer with Cu Kα radiation (λ = 1.5406 Å). The scans were performed over a 2θ range of 5–50°, with a step size of 0.02° and a 1-s acquisition time.

The simulated XRD patterns for ZIF-8 and SiO_2_ were obtained as follows: the.cif files were downloaded from single-crystal diffractometer data available at https://www.ccdc.cam.ac.uk/ (CCDC code 947064 for ZIF-8 and ICSD code 29122 for SiO_2_). These.cif files were opened using the Mercury software, and the simulated diffractograms were exported as.xy files, which were then used as simulated XRD reference patterns.

The photophysical properties were investigated through ultraviolet–visible-near-infrared (UV–vis-NIR) absorption and photoluminescence spectroscopy. UV–vis spectra were recorded on a Shimadzu UV-2600*i*/2700i spectrometer. Photoluminescence spectra were acquired using a Horiba Scientific Fluorolog-3 FL3–22TAU3 system equipped with a continuous 450 W xenon lamp and a UV xenon flash tube for excitation. A double-grating monochromator was used for both the excitation and emission. The emission and excitation spectra were corrected for the system’s wavelength response and lamp intensity profile using correction factors provided by the manufacturer. The 5D_0_ lifetime (τ_exp_) of EuTTA was measured by monitoring the 5D_0_ → 7F_2_ transition under excitation at λ_ex_ = 397 nm.

### 2.4 Adsorption–Desorption Assay

#### 2.4.1 Calibration Curve for Uvaol

UV–vis spectroscopy was employed to quantify uvaol released from the SiO_2_/EuTTA/ZIF-8 composite. Calibration curves were constructed by analyzing uvaol concentrations in the range of 0–20 μM in phosphate-buffered saline (PBS, pH 7.4) and monitoring the absorption at 216 nm, as shown in Figure S1.

#### 2.4.2 Drug Loading Assay (SiO_2_/EuTTA/ZIF-8-Uvaol)

Uvaol loading was conducted by exposing 2 mg of the SiO_2_/EuTTA/ZIF-8 nanocomposite, predried at 80 °C for 24 h, to 1 mL of a 2.2 mM uvaol solution in PBS. The samples were agitated for 24 or 72 h to promote adsorption. Subsequently, the samples were centrifuged at 13,000 × g for 10 min and air-dried at room temperature.^23^

The drug-loading capacity (DLC) was calculated using Equation 1.^24^

$$\mathrm{DLC}\;\left(\%\right)=\frac{\left[(\mathrm{mass of uvaol added})-(\mathrm{mass of uvaol in the supernatant})\right]}{\left(\mathrm{mass of}\;{\mathrm{SiO}}_{2}/\mathrm{EuTTA}/\mathrm{ZIF}-8\;\mathrm{before loading}\right)}\times 100\%$$ 1

#### 2.4.3 Drug Release Assay

The drug release experiments were carried out at 37 °C in PBS (pH = 7.4), with the temperature maintained using a water bath. For each time point, 2 mg of the SiO_2_/EuTTA/ZIF-8-uvaol nanocomposite was added to 1 mL of PBS in a glass vial. The mixture was first dispersed using ultrasonic treatment for 10 min to ensure homogeneous distribution, and then kept under constant stirring, protected from direct light, for the required incubation periods (0, 2, 4, 8, 24, and 48 h).

To maintain sink conditions, an individual vial was used for each time point (batch release method), with solids taken from the same preparation batch. After each incubation period, the samples were centrifuged at 13,000 × g for 10 min. After each release time, the supernatant was collected and separated by centrifugation, and the uvaol concentration was quantified using UV–vis spectrophotometry at 216 nm. All experiments were performed in triplicates.

Two kinetic models were used to analyze the *in vitro* drug release data. The pseudo-first-order model is used to describe systems where the rate of drug release is dependent on the drug concentration.^23^ The Korsmeyer-Peppas model is applied to describe drug release from polymeric systems, particularly when the release follows a diffusion-controlled process.^24^

The pseudo-first-order equation is given by

$$F=M_{t}/M_{\infty }=1-e^{-K\cdot t}$$ 2

where *M_t_*/*M*_∞_ is the fraction of drug released at time *t* and *k*_H_ is a constant reflecting the design variables of the system.

The Korsmeyer–Peppas equation is expressed as

$$F=M_{t}/M_{\infty }={K\cdot t}^{n}$$ 3

where *M_t_*/*M*_∞_ is the fraction of drug released at time *t*, *K* is the rate constant, and *n* is the release exponent, which characterizes the release mechanism.

### 2.5 Molecular Docking

Molecular docking calculations were performed to determine how uvaol interacted with ZIF-8. The initial structure of uvaol was built using Avogadro, an open-source molecular builder and visualization tool, version 1.2,^25^ and its structure of ZIF-8 was retrieved from the CCDC (Cambridge Crystallographic Data Centre) structural database.^26^ Subsequently, both structures were optimized using the semiempirical quantum method PM6.^27^ This method was chosen based on a previous study that evaluated the accuracy of available methods in reproducing the crystallographic structure of ZIF-8. The atomic partial charges for both structures, along with Cartesian coordinates, were obtained from calculations using the PM6 method. PM6 calculations were performed using the MOPAC 2016 program^28^ with the following keywords: Hamiltonian - PM6, gradient normalization-GNORM = 1, and defining the number of fundamental unit cells in each dimension: MERS = (1,1,1).

A grid box of 44 × 44 × 44 Å with a grid point spacing of 0.375 Å was centered on one of the potential interaction sites. The parameters x, y, and z, representing the box position, were 17.869, 4.227, and 14.316, respectively. This box dimension was sufficient to encompass the suspected interaction region of the macromolecule with the uvaol molecule. During the docking simulation, the ZIF-8 molecule remained completely rigid, while the uvaol molecule was flexible in terms of translation and orientation relative to ZIF-8. The Lamarckian Genetic Algorithm (LGA)^29,30^ was used in the calculation, with an initial population of 100 random individuals. The conformations with the lowest energy were docked and ranked in ascending order of energy according to a root-mean-square deviation (RMSD) of less than 2 Å. The docking calculations were performed using the computational package Autodock 4.2, and the grid maps used to determine the total interaction energy between the molecules were calculated using AutoGrid4 software.^31^

### 2.6 *In Vitro* Assays

#### 2.6.1 Cell Culture

The J774 murine macrophage cell line and A549 human lung epithelial cell line were provided by the Cell Bank of Rio de Janeiro. The cells were maintained in DMEM media supplemented with 10% of heat-inactivated fetal bovine serum (FBS), l-glutamine (2 mM), and gentamicin (40 μg/mL) at 37 °C and in a humidified atmosphere containing 5% CO_2_. Assays were performed using cells between three and six passages. In all experiments, untreated cells were used as negative controls.

#### 2.6.2 Cellular Viability Assay

MTT assay was used to evaluate whether uvaol, SiO_2_/EuTTA/ZIF-8, or the complex (SiO_2_/EuTTA/ZIF-8-uvaol) affected cell viability. Briefly, J774 macrophages (5 × 10^3^/well) and A549 cells (10^4^ cells/well) were seeded in 96-well plates and treated with uvaol, SiO_2_/EuTTA/ZIF-8, and SiO_2_/EuTTA/ZIF-8-uvaol at concentrations of 0.1, 1, 10, or 100 μg/mL for 24 h. Cell maintained in DMEM and treated with PBS served as control. Following this period, the medium was replaced with fresh medium containing 5 mg/mL 3-(4,5-dimethylthiazol-2-yl)-2,5-diphe-nyltetrazolium bromide). Following incubation (4 h) in a humidified CO_2_ incubator at 37 °C and 5% CO_2_, the supernatant was discarded and dimethyl sulfoxide solution (DMSO, 150 μL/well) was added to each well to solubilize the formazan crystals that had formed. After incubation at room temperature for 15 min, optical density (O.D.) was measured at 540 nm using a spectrophotometer. Three separate wells were used for each treatment. The percentage viability relative to the control sample was determined as (O.D. of treated cells/O. D. for untreated cells) × 100 cells.

#### 2.6.3 Lipopolysaccharide (LPS)-Stimulated Cytokines Secretion

For cytokine secretion stimulation, J774 macrophages were plated at a density of 7 × 10^4^ cells/mL in 48-well plates and incubated for 24 h. The cells were then treated with uvaol, SiO_2_/EuTTA/ZIF-8, and SiO_2_/EuTTA/ZIF-8-uvaol at 0.1, 1, and 10 μg/mL for 4 h. Subsequently, LPS was added at a final concentration of 20 μg/mL, and the cells were incubated for an additional 24 h. Following the stimulation, the supernatant was collected by centrifugation (2555 × g for 40 min at 4 °C), and concentrations of the cytokines TNF-α and IL-6 were quantified using enzyme-linked immunosorbent assay (ELISA), following the manufacturer’s instructions.

#### 2.6.4 Induction and Evaluation of Epithelial-Mesenchymal Transition (EMT) in A549 Cells by TGF-β1 and Cell Morphology Analysis

Epithelial-mesenchymal transition (EMT) in A549 epithelial cells was initially evaluated through morphological analysis. For induction of EMT, A549 cells at a density of 5 × 10^4^ cells/ml were seeded with TGF-β1 at 10 ng/mL in DMEM medium containing 5% FBS, 100 U/ml penicillin, and 100 mg/mL streptomycin and incubated for 24 h at 37 °C in an atmosphere of 5% CO_2_. To determine the effect of uvaol and its nanocomposite on EMT, A549 cells were seeded in 24-well plates and incubated overnight. After 24 h, the cells were treated with uvaol or SiO2@EuTTA@ZIF-8-uvaol (0.1 μg/mL), followed by stimulation with TGF-β1 (5 ng/mL). After 24 h, the cells were fixed with methanol for 10 min, stained with crystal violet, and observed under an inverted phase-contrast microscope. Photomicrographs were randomly captured and morphological characteristics related to TEM were evaluated. Cells stained with crystal violet were evaluated under a light microscope (Nikon, Japan), and images were acquired using image analysis software (NIS-Element, Nikon, Japan). Morphological parameters such as “circularity,” “roundness,” and “aspect ratio” were quantitated from the images using the shape descriptor parameters described by Zanier et al.^32^

#### 2.6.5 Scratch Wound Healing Assay

Twenty-thousand A549 cells per well were seeded into 24-well plates and maintained until they reached 90% confluence. The cells were treated with 3 μg/mL mitomycin C (Sigma-Aldrich) for 3 h. Monolayers were gently and slowly scratched with a 200 μL pipet tip across the center of the well. After washing with PBS twice, the cells were treated in the absence or presence of TGF-β1 alone (5 ng/mL) or in combination with 0.1 μg/mL uvaol and/or SiO_2_@EuTTA@ZIF-8-uvaol for 24 h. At the end of the treatment period, images were acquired from each well using an Olympus BX51 microscope coupled with a digital camera at 0 and 24 h. Cell migration was analyzed using the ImageJ software, and gap closure was expressed as a percentage (%) of the control. The values were obtained through the following calculation: wound closure (%) = [(A0- At)/A0] × 100. where A0 is the area at time 0, and At is the area after the gap.

#### 2.6.6 Statistical Analysis

Data are reported as the mean ± standard error of the mean (S.E.M.) and were analyzed using GraphPad Prism software (version 5.0 (San Diego, CA, USA). Comparisons between the experimental groups were performed using one-way analysis of variance (ANOVA) followed by Tukey’s HSD test or two-way ANOVA followed by the Bonferroni test. Statistical significance was set at *p* < 0.05.

## 3 Results and Discussion

### 3.1 Structural and Morphological Properties

X-ray diffraction (XRD) analysis was initially used to confirm the crystallographic phase and verify the purity of ZIF-8. As shown in Figure 1, the X-ray diffractogram demonstrated good agreement between the experimental diffraction peaks and the simulated ZIF-8 pattern derived from the CIF file (CCDC 947064), available from the Cambridge Crystallographic Data Centre.^33^ No additional peaks were observed, which suggests that the method employed for synthesizing the coordination polymers effectively produced high-purity compounds.

**Figure 1:**
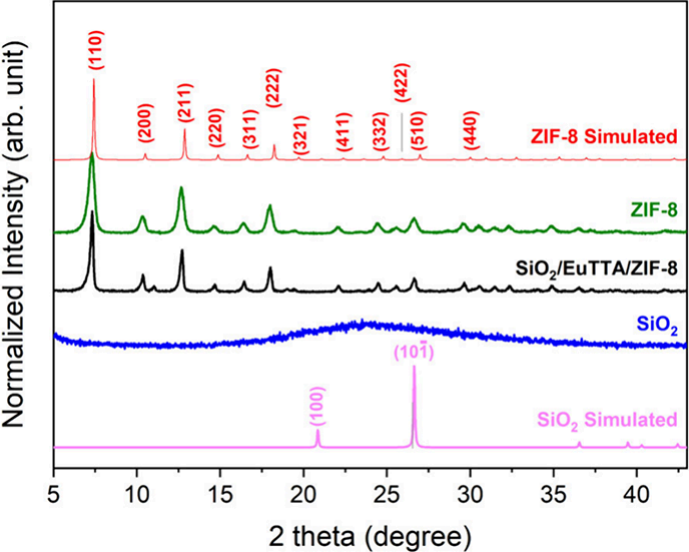
X-ray diffraction patterns of synthesized materials. Powder X-ray diffraction patterns are shown for ZIF-8, SiO_2_, SiO_2_/EuTTA/ZIF-8, and the simulated patterns of ZIF-8 and SiO_2_ obtained from crystallographic data (CCDC 947064 and ICSD 29122, respectively).

XRD measurements were also conducted for SiO_2_ and SiO_2_/EuTTA/ZIF-8 (Figure 1). The X-ray diffraction pattern of SiO_2_ exhibited a broad amorphous halo between 15° and 30°, a characteristic feature indicative of silica formation, typically associated with the (100) and (10–1) planes.^25,34^ Additionally, the XRD pattern of SiO2/EuTTA/ZIF-8 showed the characteristic diffraction peaks of ZIF-8, confirming that the coordinating compound was successfully incorporated into the luminescent silica.

To investigate the morphological properties of the materials, SiO_2_, ZIF-8, SiO_2_/EuTTA, and SiO_2_/EuTTA/ZIF-8 samples were analyzed using Scanning Electron Microscopy (SEM) (Figure 2). SEM analysis revealed that the SiO_2_ particles were spherical with an average size of 32 ± 7 nm (Figure 2A and S2A). The ZIF-8 micrograph (Figures 2B and S2B) displays the coordination polymer as small irregular blocks arranged in conglomerates, a morphology consistent with that described in the literature,^21^ with an average particle size of 111 ± 30 nm. The synthesis of SiO_2_/EuTTA (Figures 2C and S2C) resulted in irregular nanoparticles with spherical morphology and an average particle size of 90 ± 20 nm. Finally, the SiO_2_/EuTTA/ZIF-8 composite (Figures 2D and S2D) was characterized by spherical nanoparticles with an average size of 108 ± 35 nm. Notably, the particle size of SiO_2_/EuTTA/ZIF-8 falls within the nanoscale range, which is crucial for enhancing its potential as a biomaterial.

**Figure 2:**
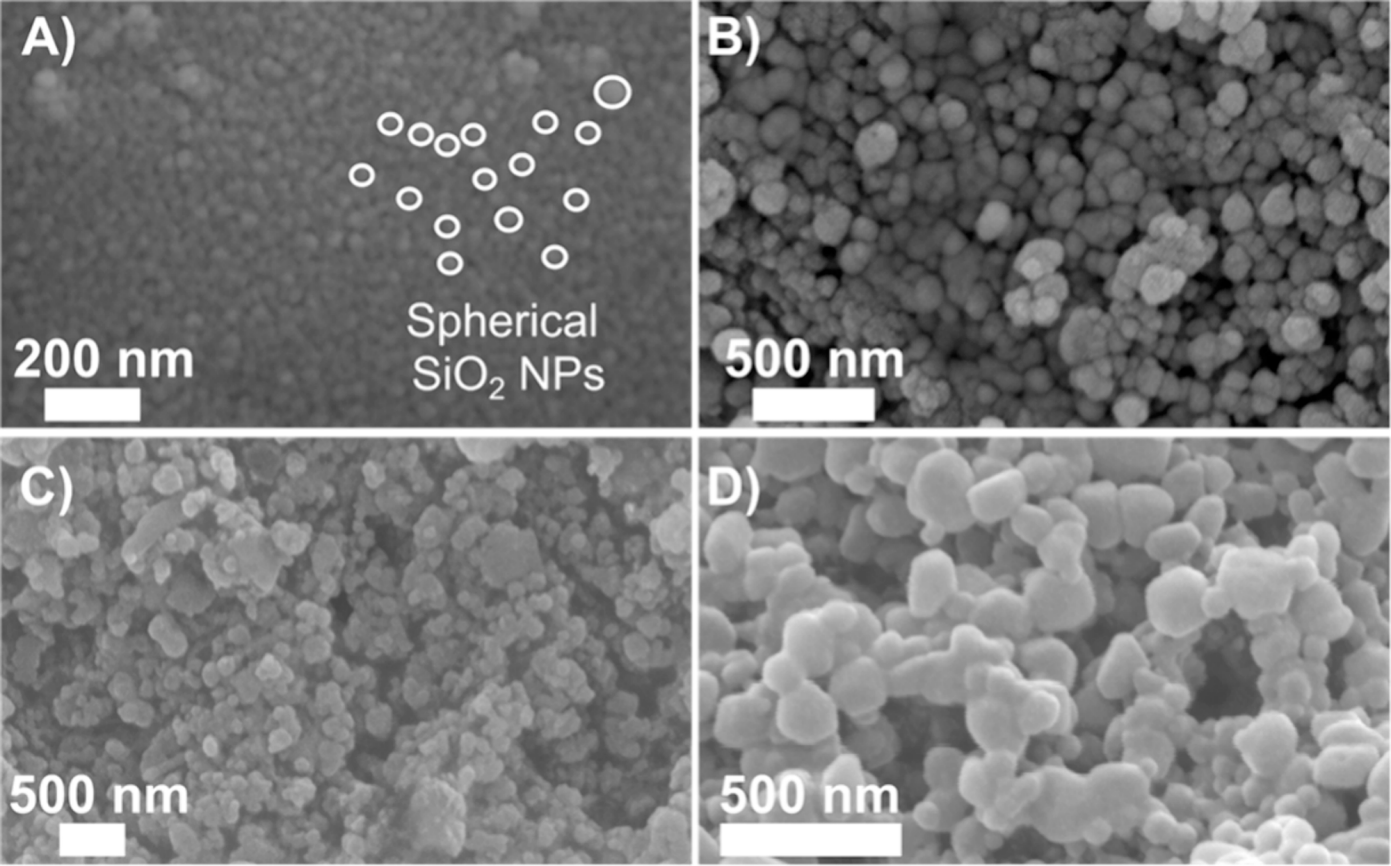
Morphological analysis of synthesized nanoparticles via SEM. SEM micrographs are shown for (A) SiO_2_, (B) ZIF-8, (C) SiO_2_/EuTTA, and (D) SiO_2_/EuTTA/ZIF-8.

### 3.2 Chemical and Thermal Analysis

For the chemical characterization of the ZIF-8, EuTTA, SiO_2_, SiO_2_/EuTTA, and SiO_2_/EuTTA/ZIF-8 compounds, vibrational spectroscopy in the infrared region (FT-IR) was employed (Figure 3).

**Figure 3:**
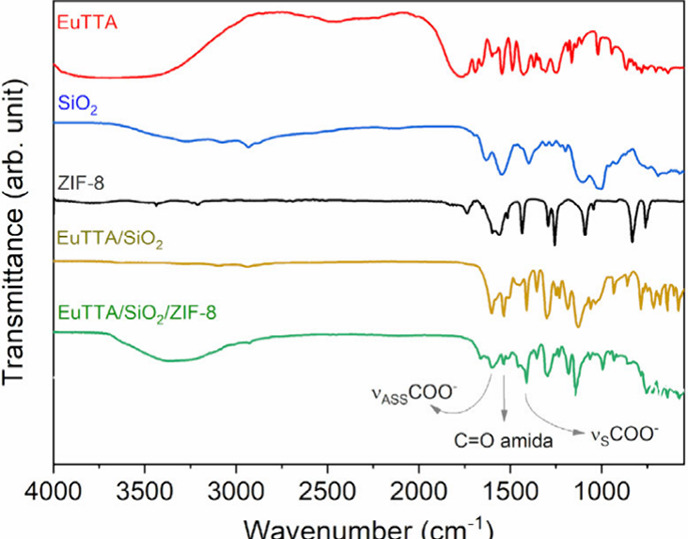
Chemical analysis of the synthesized materials was performed using FT-IR spectroscopy. FT-IR spectra are shown for EuTTA (red line), SiO_2_ (blue line), ZIF-8 (black line), SiO_2_/EuTTA (yellow line), and SiO_2_/EuTTA/ZIF-8 (green line).

The SiO_2_ particles were characterized by the presence of a Si–O–Si stretching band centered at 1070 cm^–1^, indicating the condensation between the silane groups (Figure 3). Additionally, two signals were observed at 1635 cm^–1^ and 1700 cm^–1^, corresponding to the amide and carboxylic acid groups, respectively, confirming the success of the functionalization process. Figure S3 illustrates the reaction mechanism and shows the correct functionalization of the particles during their synthesis. This functionalization is crucial for complexation of the EuTTA complex in the nanocomposite material.

The EuTTA complex (Figure 3) was characterized by a broad band with a maximum centered at 1769 cm^–1^, attributed to the C=O stretching of the ketone group of the coordinated HTTA ligand, as described in the literature.^35,36^ FT-IR analysis of ZIF-8 (Figure 3) revealed vibrations centered at 1568 cm^–1^, assigned to the C=N stretching vibration of the imidazole ring, as well as signals in the region between 1520 and 1290 cm^–1^, corresponding to the total stretching of the imidazole ring (C–N symmetric stretching). Additionally, bands between 1220 and 930 cm^–1^ were observed, indicating torsion in the ring (C–N asymmetric stretching).^35,36^

The functionality of the silica with the coordination compound in SiO_2_/EuTTA was confirmed by the asymmetric and symmetric COO^–^ stretches at 1601 cm^–1^ and 1403 cm^–1^, respectively (Figure 3). The presence of silica in the system was also verified by the signal at 1635 cm-^1^, corresponding to the C=O group of the amide group.^36,37^

As shown in Figure 3 (green line), the characterization of the ZIF-8 signals in the SiO_2_/EuTTA/ZIF-8 compound was hindered by the overlap of the signals from the other materials. However, slight changes in the band profile were still noticeable, particularly in the region between 1500 and 1400 cm^–1^, attributed to the total stretching of the imidazole ring (C–N symmetric stretch).

Figure 4 illustrates the thermal characterization of ZIF-8 (black line) and SiO_2_/EuTTA/ZIF-8 (blue line) using thermogravimetric analysis (TGA). The ZIF-8 thermogram showed a gradual 9% mass loss between 50 and 250 °C, followed by the elimination of the binder in two stages between 200 and 900 °C, resulting in a mass loss of 64%. The residual mass of 27% corresponds to the formation of ZnO, which is consistent with the literature data for ZIF-8.^37^

**Figure 4:**
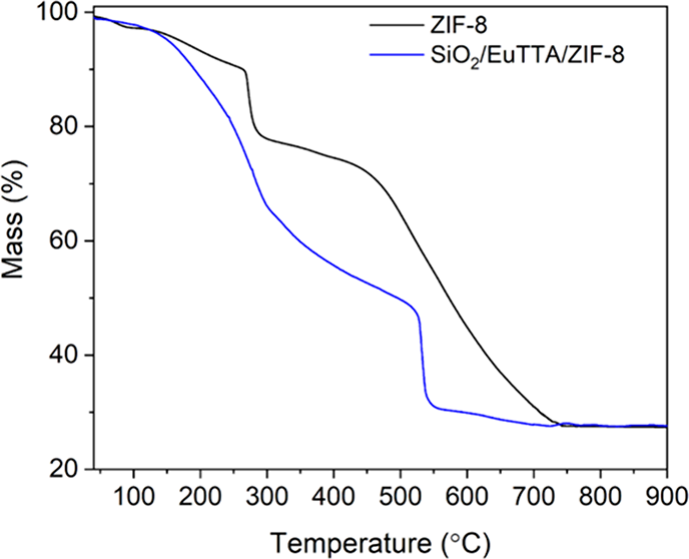
Thermal stability analysis by TGA. Thermograms are shown for compounds ZIF-8 (black line) and SiO_2_/EuTTA/ZIF-8 (blue line).

The TGA curve for the SiO_2_/EuTTA/ZIF-8 nanocomposite displays a different thermal behavior compared to ZIF-8 (Figure 4). Three new mass loss events were observed in the temperature ranges of 50–350 °C, 350–560 °C, and 360–800 °C, corresponding to mass losses of 40%, 29%, and 3%, respectively. This abrupt change in the thermogram is primarily attributed to sublimation of the EuTTA complex, a process commonly observed for lanthanide complexes with β-diketonate ligands.^38,39^ As a result, the SiO_2_/EuTTA/ZIF-8 nanocomposite exhibited a residual mass of 2%, which is close to the 27% residual mass found for ZIF-8.

### 3.3 Photoluminescence Properties

The luminescence properties of the EuTTA compound were evaluated through the excitation (λem = 611 nm) and emission (λex = 397 nm) spectra, as shown in Figure S4. In the excitation spectrum, a broad band is observed in the 250–500 cm^–1^ region, corresponding to the π → π* transitions of the coordinated ligand. Additionally, the presence of the 5D_2_ ← 7F_4_ transition from the Eu^3+^ ion is evident. The emission spectrum of EuTTA revealed the characteristic transitions 5D_0_ → 7F_J_ (where J = 0, 1, 2, 3, and 4) of the trivalent Eu ion. Notably, the optical behavior observed for the EuTTA complex closely resembles that reported in the literature,^22^ confirming that the desired complex was successfully synthesized. The decay curve of EuTTA was also recorded (Figure S5). The decay profile was best fitted using a monoexponential equation, yielding a lifetime of 0.22 ± 0.01 ms.

The optical properties of SiO_2_/EuTTA and SiO_2_/EuTTA/ZIF-8 were also analyzed to verify the preservation of photoluminescence exhibited by the EuTTA complex (Figure 5). Taking advantage of the europium ion (III) as a structural probe, we examined the emission spectra of these materials to detect any potential changes upon formation of the composite material (λex = 397 nm). The spectra exhibited significant similarity to one another, indicating that the symmetry of Eu^3+^ in the coordination compound remained largely unaltered upon incorporation into the nanocomposite material.

**Figure 5:**
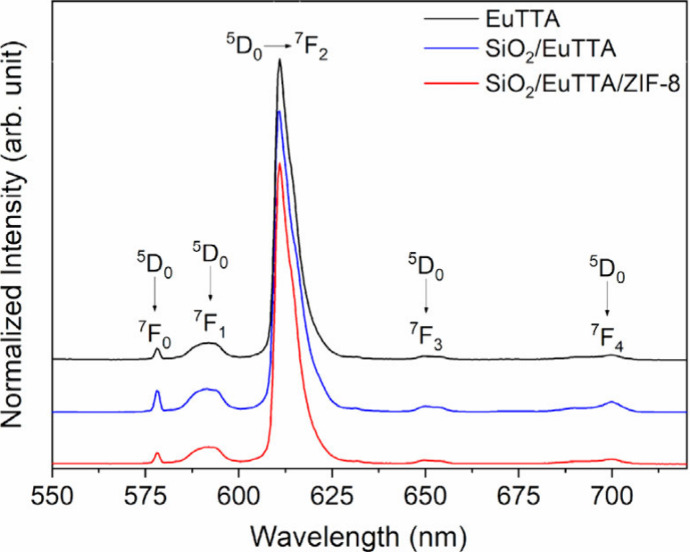
Analysis of photoluminescent properties in the nanocomposite. Emission spectra (λ_Ex_ = 397 nm) are shown for EuTTA compound (black line), SiO_2_/EuTTA (blue line), and SiO_2_/EuTTA/ZIF-8 (red line)

### 3.4 Uvaol Loading and Release in SiO_2_/EuTTA/ZIF-8

UV–visible absorption spectroscopy was employed to assess the effective storage capacity of uvaol at a concentration of 20 μM (Figure S1). A broad and pronounced absorption band in the region of 200–240 nm was observed, which closely matched the data reported in the literature.^31^ This UV–vis spectrum was used as the standard for constructing a calibration curve (y = 0.02 + 0.08x, R^2^ = 0.999), which was applied to quantify the adsorbed material in the luminescent nanocomposite.

The loading of uvaol onto SiO_2_/EuTTA/ZIF-8 was achieved by exposing 2 mg of the SiO/EuTTA/ZIF-8 nanocomposite to 2.2 μmol of uvaol in PBS buffer (pH 7.4), with adsorption times evaluated at 24 and 72 h, as shown in Figure 6. Results indicated that adsorption efficiency depended on the exposure duration, with 27% (0.59 μmol of uvaol) and 68% (1.50 μmol of uvaol) adsorbed for 24 and 72 h, respectively.

**Figure 6:**
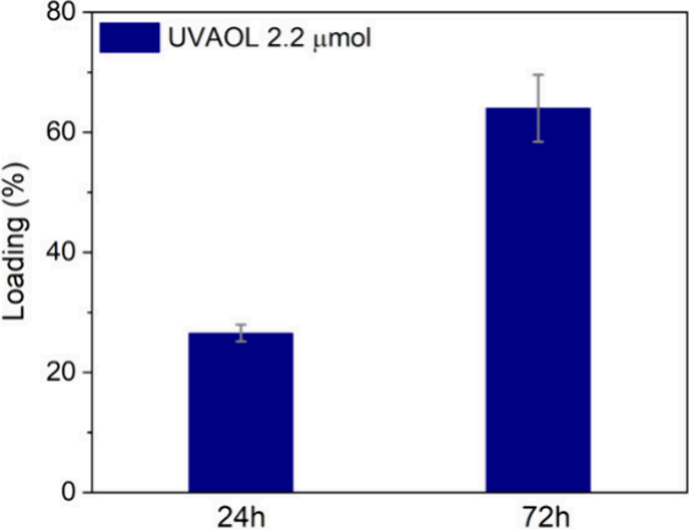
Uvaol loading efficiency on SiO_2_/EuTTA/ZIF-8. Percentage of uvaol loading on the SiO_2_/EuTTA/ZIF-8 nanocomposite is shown as a function of the added amount of uvaol, evaluated at adsorption times of 24 and 72 h. The bars represent the mean ± standard error of the mean (S.E.M.) of three experiments performed in triplicate.

It is important to note that a 68% loading efficiency is considered very high according to the literature, especially for ZIF-8-based drug delivery systems.^40–42^ Thus, using 2.2 μmol of uvaol over 72 h was selected as the most efficient protocol for further experiments. From the adsorption studies, the drug loading capacity (DLC) was calculated to be 0.33 mg of uvaol per mg of SiO_2_/EuTTA/ZIF-8. This loading efficiency is comparable to, and in some cases surpasses, the values reported in the literature for ZIF-8-based drug delivery systems, such as doxorubicin-ZIF-8, which has a DLC of 0.049.^23^ These findings highlight the potential of the SiO_2_/EuTTA/ZIF-8-uvaol nanocomposite as a promising platform for drug delivery.

As shown in Figure 7, a drug release study was conducted to evaluate the release kinetics of uvaol adsorbed onto the SiO_2_/EuTTA/ZIF-8 nanocomposite in PBS buffer (pH 7.4), offering insights into its potential as a sustained drug delivery system. Initial results indicated a rapid release phase, with approximately 52% of the uvaol content released within the first 8 h, suggesting effective desorption under physiological pH conditions. Following this initial burst, a more controlled and stable linear release profile was observed, which persisted over the duration of the experiment, implying a continuous release mechanism likely supported by the composite’s nanostructured porosity and surface interactions. The cumulative release appeared to stabilize between 10 and 48 h, suggesting that the system may have reached a release equilibrium. This behavior is consistent with previous reports of partial release in MOF-based and mesoporous systems, where strong drug–matrix interactions or entrapment can limit the overall release.^43–45^

**Figure 7:**
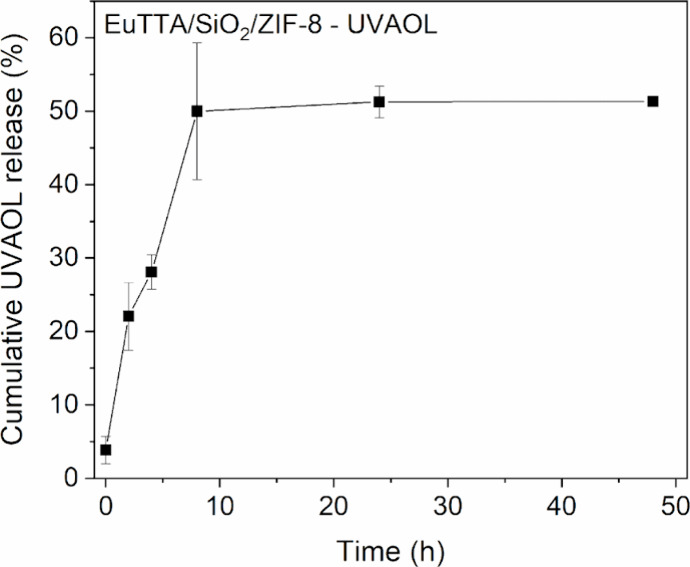
Drug release profile of SiO_2_/EuTTA/ZIF-8-uvaol. Drug release profile of the SiO_2_/EuTTA/ZIF-8-uvaol nanocomposite in PBS (pH = 7.4) is shown over a 48-h period. Each data point (■) represents the mean ± standard error of the mean (S.E.M.) of three experiments performed in triplicate.

This biphasic release profile, encompassing a rapid initial phase followed by sustained release, aligns with the desired characteristics of drug delivery systems, in which an initial burst delivers a therapeutic dose quickly, followed by slower release to maintain drug levels over time. These results suggest that the SiO_2_/EuTTA/ZIF-8 framework offers both efficient drug encapsulation and controlled release potential, making it suitable for applications that benefit from steady drug concentrations, such as chronic inflammatory conditions. Future studies could explore the effects of varying pH levels and buffer compositions to assess the stability and adaptability of the release profile in diverse biological environments.

The desorption of uvaol from the SiO_2_/EuTTA/ZIF-8 nanocomposite was evaluated using different kinetic models to gain insights into the release mechanism. Two commonly applied models were used for this purpose: the pseudo-first-order model and Korsmeyer-Peppas model. The fitting results of both the models are summarized in Table 1, and the corresponding desorption curves are shown in Figure S6.

**Table 1:** Interpretation of R-Square Values and Rate Constants of the Release Kinetics of SiO_2_/EuTTA/ZIF-8-Uvaol Nanoparticles at pH 7.4

Model	*R* ^2^	*k*	*n*
Pseudo-first-order	0.992	0.21	-
Korsmeyer–Peppas	0.936	24.1	0.22

For the pseudo-first-order model, a high correlation coefficient (R^2^ = 0.992) was obtained, indicating that uvaol release may be governed by a first-order kinetic process. However, the rate constant (K) for the pseudo-first-order model was calculated to be 0.21, suggesting a relatively slow desorption process, which is indicative of the controlled and gradual release of uvaol from the nanocomposite.

In contrast, the Korsmeyer-Peppas model, which accounts for both Fickian and non-Fickian diffusion mechanisms, yielded a lower correlation coefficient (R^2^ = 0.936) and a significantly higher release rate constant (K = 24.1). The value of n in this model is 0.22, which is consistent with the behavior of a non-Fickian diffusion mechanism. This model suggests that the desorption of uvaol from the SiO_2_/EuTTA/ZIF-8 nanocomposite is likely governed by a combination of diffusion and relaxation processes,^24,46^ in which the release is not purely governed by diffusion, but also involves the structural rearrangement of the nanocomposite and interactions between the uvaol molecules and the nanocomposite.

Comparing the two models, the pseudo-first-order model provides a better fit in terms of the correlation coefficient, indicating that the desorption of uvaol may primarily follow a first-order release process. However, despite the slightly lower R^2^ value, the Korsmeyer-Peppas model provides valuable insights into the nature of the release mechanism, particularly in terms of the n value. An n value of 0.22 indicates that the release mechanism is closer to a diffusion-controlled process, with a slight deviation toward non-Fickian behavior.

In practical terms, the results suggest that the EuTTA/ZIF-8 nanocomposite exhibited a controlled release profile in which uvaol was gradually desorbed over time. The mechanism is likely dominated by diffusion and possibly by some structural relaxation of the nanocomposite. This controlled release could be advantageous for drug delivery applications by ensuring sustained and predictable release rates.

Furthermore, the ability of the Korsmeyer-Peppas model to capture the dual nature of the release mechanism (involving both diffusion and relaxation) may indicate the role of the ZIF-8 porous structure in modulating the release rate. The organization of the nanocomposite appears to facilitate slow, sustained release, which is ideal for controlled drug delivery systems.

### 3.5 Molecular Docking

Considering that the uvaol ligand underwent flexibility treatment for angles and dihedrals, multiple conformations were explored during conformational sampling and energy calculations. In total, 100 conformations were evaluated and ranked according to their interaction energies. These conformations were grouped into a bar graph based on the average overlap error, as shown in Figure 8.

**Figure 8:**
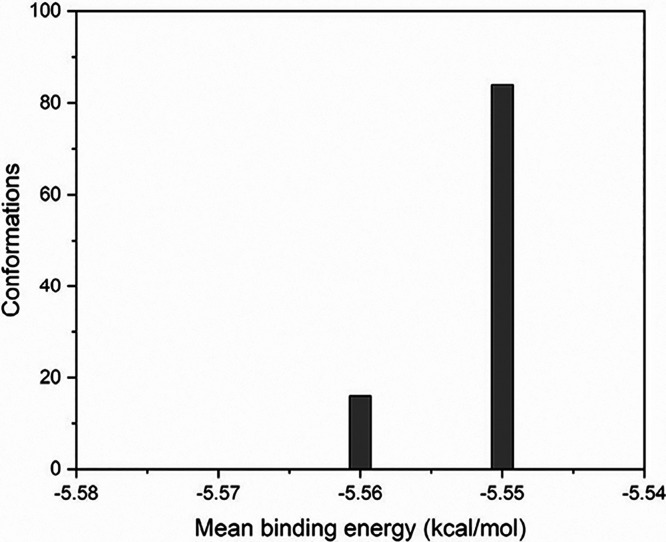
Molecular docking analysis of uvaol interactions with ZIF-8. Data is shown as a bar graph depicting grouping of different conformations simulated in the interaction of uvaol in ZIF-8.

Analysis of Figure 8 shows that 84% of the structures exhibited similar conformations, with an overlap error below 2 Å and an average binding energy of −5.55 kcal/mol. Notably, the conformation with the lowest energy within this group had an interaction energy of −5.57 kcal/mol. A smaller subset of conformations, representing 16% of the total, was obtained, with an average binding energy of −5.56 kcal/mol. Given the high percentage of conformations clustered within specific regions and low calculated energy, it is assumed that these conformations represent the most probable modes of interaction between uvaol and ZIF-8. Figure 9 illustrates the likely mode of interaction between the evaluated systems derived from the molecular docking calculations, along with the overlay of the 84 lower-energy conformations.

**Figure 9:**
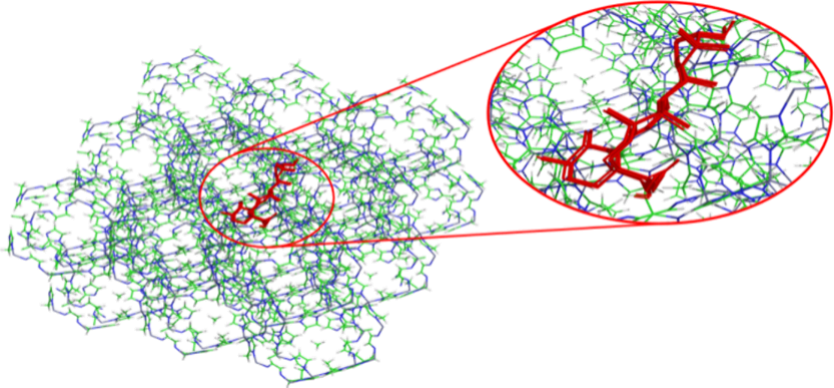
Predicted binding modes from molecular docking. Overlay of the 84 conformations clustered in the docking calculations, showing the most probable binding modes.

Based on the intrinsic characteristics of the evaluated systems (ZIF-8/uvaol) and comparison with the results reported in the literature,^47,48^ it is surprising that ZIF-8 can incorporate uvaol into its pores. Specifically, ZIF-8 has a pore entrance size of 3.45 Å, whereas the smallest dimension of the uvaol molecule, when evaluated in three dimensions, is 5.81 Å. Therefore, even considering the smallest dimension of uvaol, its incorporation into the pore seems unlikely because of significant size mismatch. However, the possibility of interaction with the ZIF-8 surface cannot be ruled out. These interactions may include hydrogen bonding with hydroxyl groups and hydrophobic interactions, as both the ZIF-8 and uvaol molecules are hydrophobic and π-π stacking interactions, as shown in Figure 10. It is important to note that the conformation used to illustrate these interactions corresponded to the lowest energy conformation within 84% of the conformations analyzed.

**Figure 10:**
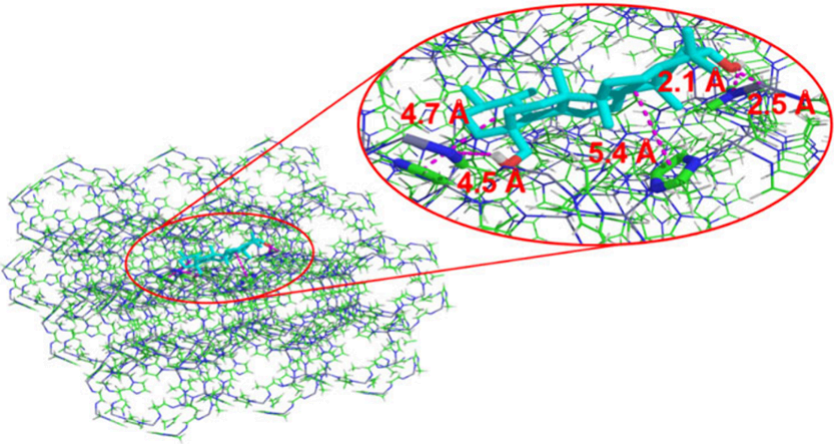
Interaction distances in docking simulations. Interaction distances between the low-energy conformation of uvaol and ZIF-8 is shown, derived from the docking simulation of the 84% conformational cluster.

During the adsorption processes, in addition to hydrogen bonding, π–π stacking interactions are commonly observed. In this context, these results suggest the presence of such interactions between the five-membered rings in both the structures, with an estimated distance of 5.3 Å. To further investigate the interactions forming the 16% conformational group, the simulation shown in Figure 11 was conducted. Analysis of the data revealed that the types of interactions observed remained largely the same, with the only difference being the relative positioning of the structure with respect to the ZIF-8 surface.

**Figure 11:**
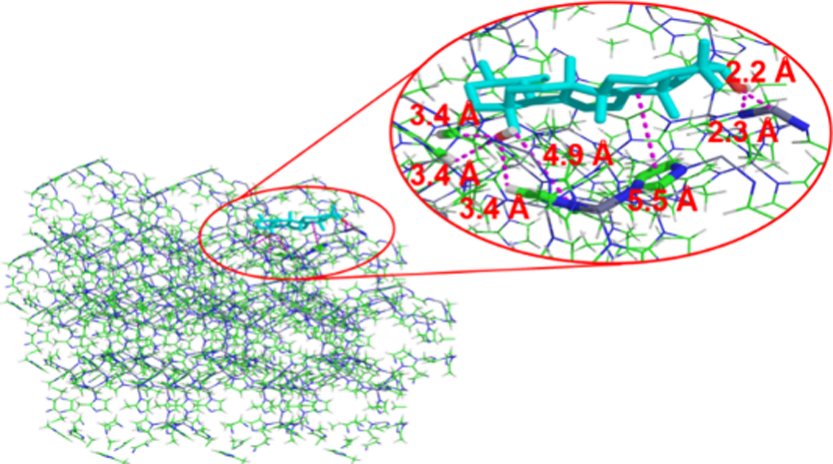
Interaction distances in docking simulations. Interaction distances between the low-energy conformation of uvaol and ZIF-8 are shown, obtained from the docking simulation of the 16% conformational grouping.

### 3.6 Effect of Uvaol, SiO_2_/EuTTA/ZIF-8, and SiO_2_/EuTTA/ZIF-8-Uvaol on Cell Viability

MTT assays were used to determine whether uvaol and its nanocomposite with SiO_2_/EuTTA/ZIF-8 affected cell viability and to ascertain the nontoxic concentration ranges of uvaol and its nanocomposite to treat cells. As shown in Figure 12, there were no significant changes in the viability of J774 macrophages and A549 epithelial cells after uvaol treatment at any of the tested doses for 24 h. However, when both cell lines were exposed to 100 μg/mL of SiO2/EuTTA/ZIF-8 and SiO2/EuTTA/ZIF-8-uvaol, there was a significant reduction in cell viability. Thus, we selected doses lower than 10 μg/mL to proceed to the next steps and evaluated their effects on other inflammatory response-related functions.

**Figure 12:**
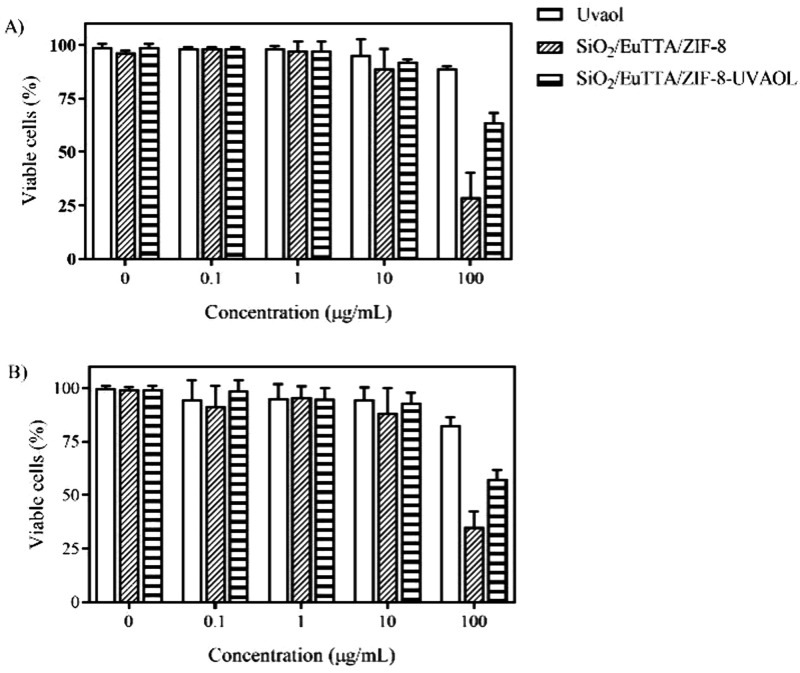
Effect of uvaol, SiO_2_/EuTTA/ZIF-8, and SiO_2_/EuTTA/ZIF-8-uvaol on cell viability. Macrophages and epithelial cells were plated and treated with uvaol (0.1–100 μg/mL) for 24 h. Cell viability was measured by MTT assay. The bars represent the mean ± standard error of the mean (S.E.M.) of three experiments performed in triplicate.

### 3.7 Effect of Uvaol, SiO_2_/EuTTA/ZIF-8, and SiO_2_/EuTTA/ZIF-8-Uvaol on Lipopolysaccharide (LPS)-Induced TNF-α and IL-6 Production of J774 Cells

To evaluate the direct effects of uvaol and its nanocomposite with SiO2/EuTTA/ZIF-8 on LPS-stimulated macrophages, J774 cells were treated with uvaol or its nanocomposite and stimulated with LPS in vitro. As shown in Figure 13A, exposure to 10 μg/mL SiO2/EuTTA/ZIF-8 did not alter the production of TNF-α, as measured in the supernatants from LPS-stimulated macrophages. However, uvaol treatment alone at 1 and 10 μg/mL significantly reduced the levels of this cytokine to 19% and 38.6%, respectively, in untreated cells.

**Figure 13:**
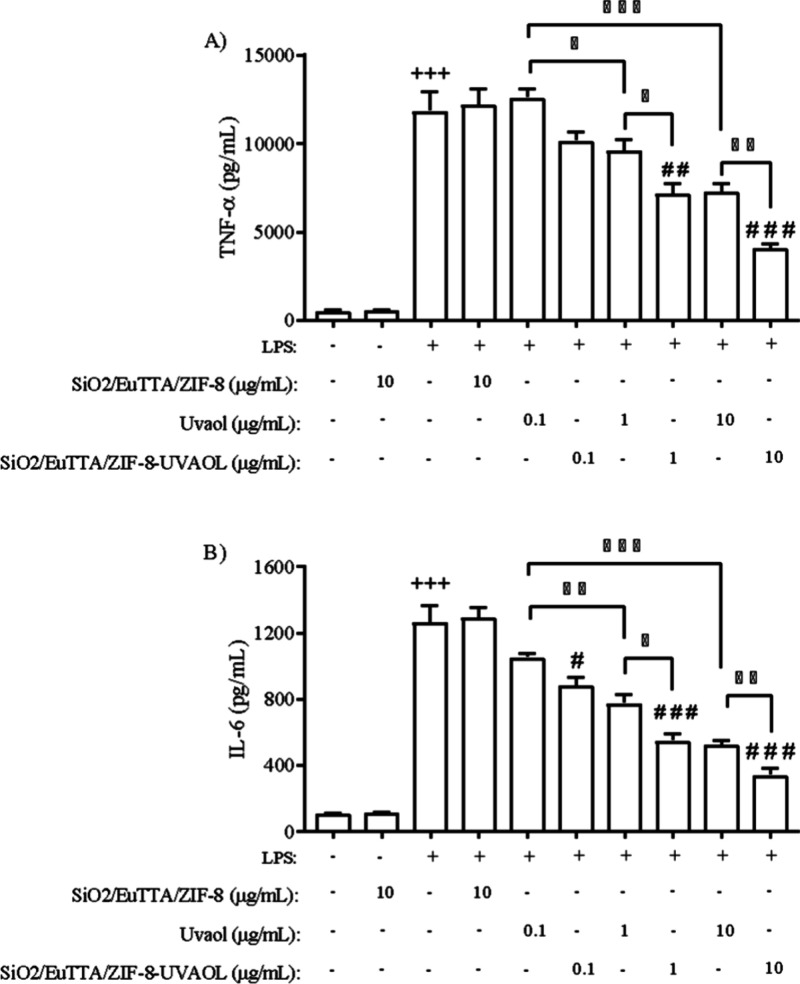
Impact of Uvaol, SiO_2_/EuTTA/ZIF-8, and SiO_2_/EuTTA/ZIF-8-uvaol on lipopolysaccharide (LPS)-induced cytokine secretion. J774 cells were pretreated for 1 h with uvaol and its nanostructures, and stimulated with LPS (20 μg/mL) for 24 h. Then, production of (A) TNF-α and (B) IL-6 were analyzed using enzyme-linked immunosorbent assay (ELISA). Bars represent means ± standard error of the mean (S.E.M.) of three independent experiments. +++*P* < 0.001 compared to unstimulated cells; # *P* < 0.05, ## *P* < 0.01 and ###*P* < 0.01 compared to LPS-stimulated cells.

Treatment with 0.1 μg/mL uvaol did not interfere with LPS-induced TNF-α secretion. However, the SiO2/EuTTA/ZIF-8-uvaol nanocomposite reduced TNF-α levels in LPS-stimulated macrophages in a dose-dependent manner. Moreover, the nanocomposite at 1 and 10 μg/mL induced inhibition even greater than that of uvaol alone. As shown in Figure 13B, uvaol complexed with SiO2/EuTTA/ZIF-8 was also more effective than uvaol alone in inhibiting the production of IL-6 in LPS-stimulated macrophages. Interestingly, treatment with 0.1 μg/mL SiO2/EuTTA/ZIF-8-uvaol significantly reduced LPS-induced IL-6 secretion, a result that uvaol alone could not accomplish.

Given that low concentration (0.1 μg/mL) significantly reduced IL-6 production and modestly attenuated TNF-α secretion following LPS stimulation, we extended the investigation evaluated its effects on epithelial-mesenchymal transition (EMT).^49^ It is important to note that the inflammatory microenvironment, composed of inflammatory cells and mediators such as IL-6 and TNF-α, induces EMT and contributes to the chronicity of several pathological conditions, including asthma.^50^ EMT is a crucial cellular trans-differentiation process implicated in chronic inflammation and tissue fibrosis, both of which contribute to the pathogenesis of airway remodeling in chronic respiratory diseases such as asthma.^51^ Indeed, epithelial-mesenchymal transition (EMT) has been described in various pulmonary diseases, including asthma, where persistent chronic inflammation leads to molecular reprogramming, resulting in aberrant tissue repair and excessive, self-sustaining profibrotic responses. Given that EMT is a key contributor to the development of fibrosis, the modulation of this cellular transdifferentiation process holds potential for preventing fibrosis progression, which is characterized by excessive extracellular matrix production and disruption of tissue architecture. In this context, the use of a nanostructure that enhances the bioavailability of a drug candidate capable of modulating EMT may represent a significant therapeutic advancement in the treatment of fibrotic diseases. Thus, we explored whether the nanocomposite form of uvaol could enhance its effects on EMT, considering the importance of optimizing its delivery and bioavailability for therapeutic applications.

### 3.8 Effect of Uvaol, SiO_2_/EuTTA/ZIF-8, and SiO_2_/EuTTA/ZIF-8-Uvaol on TGF-β1 Induced Epithelial–Mesenchymal Transition in A549 Cells

Epithelial-mesenchymal transition (EMT) is a biological process in which epithelial cells are phenotypically transformed into mesenchymal cells. Recent studies have proposed that EMT plays a central role in the pathophysiology of chronic airway diseases; therefore, so much so that this process has become a potential clinical therapeutic target because it is activated under pathological conditions.^52^

As shown in Figure 14, we explored the effects of uvaol and its nanocomposite SiO_2_/EuTTA/ZIF-8-uvaol on TGF-β1-induced epithelial-mesenchymal transition (EMT) in A549 cells. Morphological evaluation revealed that untreated A549 cells retained their epithelial cobblestone morphology, whereas TGF-β1 stimulation resulted in significant EMT-related morphological changes, including elongation, branching, and loss of epithelial phenotype. Treatment with 0.1 μg/mL uvaol alone did not reverse these EMT alterations (Figure 14A). However, cells treated with the SiO_2_/EuTTA/ZIF-8-uvaol nanocomposite exhibited a significantly reduced mesenchymal phenotype, with less elongation and a morphology closer to that of unstimulated cells (Figure 14A). Quantitative analysis further confirmed these findings, in which TGF-β1-induced EMT led to reduced cell circularity (Figure 14B) and roundness (Figure 14C). While uvaol alone did not affect these parameters, the nanocomposite significantly restored the circularity and roundness of TGF-β1-stimulated cells. Importantly, the SiO_2_/EuTTA/ZIF-8 nanocarrier alone did not induce any morphological changes in unstimulated A549 cells and did not affect TGF-β1-induced epithelial-mesenchymal transition (EMT). This result indicated that the observed effects on EMT reversal were specifically mediated by uvaol encapsulated within the nanocomposite. The inert behavior of the nanocarrier ensured that its role was limited to enhancing uvaol delivery and bioavailability without independently influencing cellular behavior or morphology. These findings underscore the specificity and effectiveness of SiO_2_/EuTTA/ZIF-8-uvaol nanocomposite in targeting TGF-β1-induced EMT.

**Figure 14:**
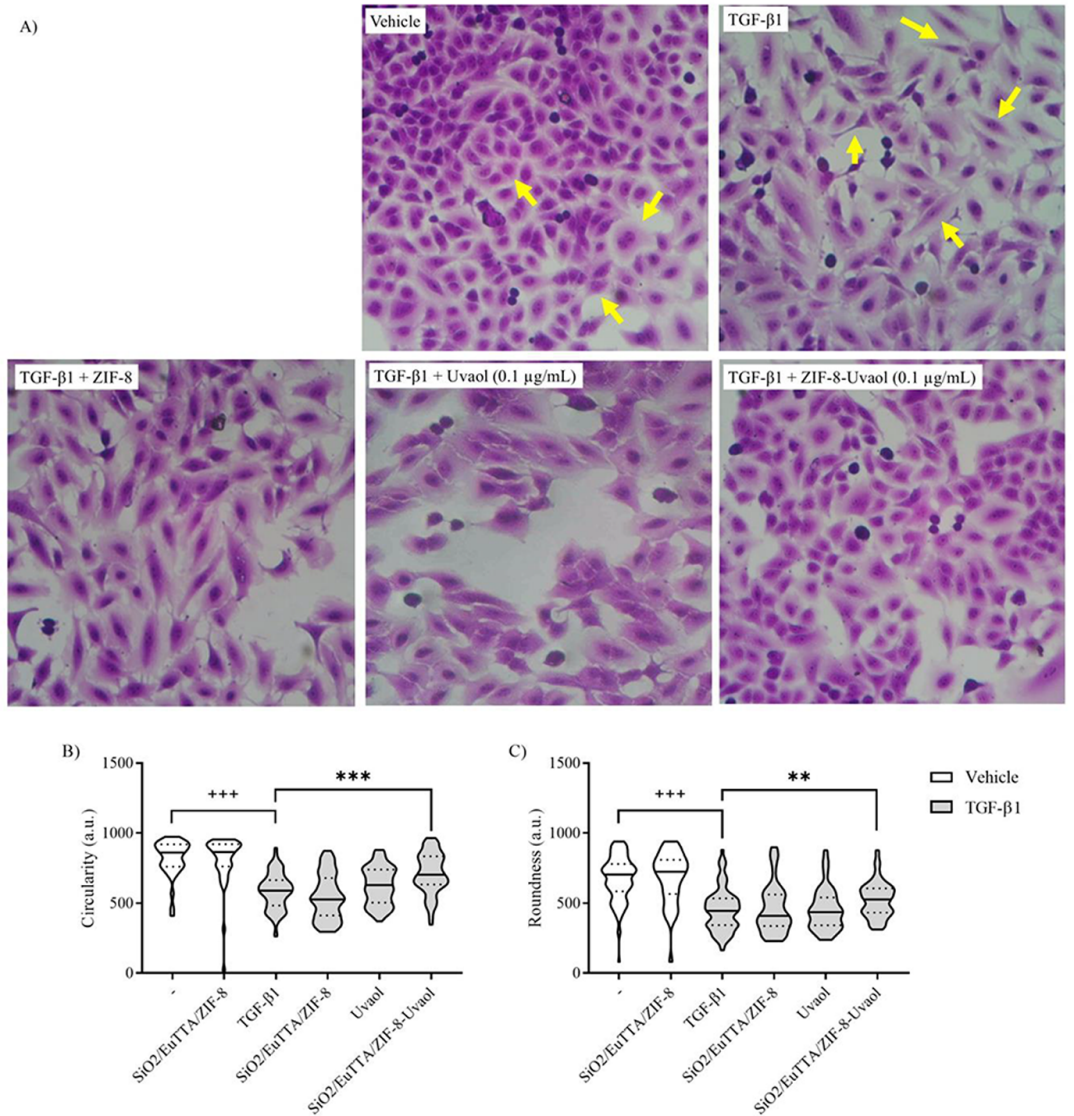
Inhibition of TGF-β1-Induced EMT in A549 Cells. The effect of uvaol, SiO_2_/EuTTA/ZIF-8, and SiO_2_/EuTTA/ZIF-8-uvaol on TGF-β1-induced morphological changes in A549 cells was assessed. Morphologic changes consistent with EMT were observed in TGF-β1-treated cells for 24 h, compared with vehicle-treated cells. Control cells (not exposed to TGF-β1 at 5 ng/mL and cultured in DMEM medium) retained the cobblestone morphology characteristic of epithelial cells (as shown by the arrows in the control panel). Phenotypic changes toward a fibroblast-like phenotype were evident in TGF-β1-treated cells as evaluated by light microscopy and crystal violet staining (panel A). Violin plots display the distribution of circularity (panel B) and roundness (panel C) in A549 cells treated with either vehicle (DMSO) alone or TGF-β1 for 24 h. Morphometric analyses were conducted using ImageJ FiJi software, and statistically significant differences between groups were identified by One-way ANOVA followed by the Kruskal–Wallis test. Data are represented as means ± standard error of the mean (S.E.M.). Statistical significance is indicated as follows: +++ *P* < 0.001 compared to unstimulated cells; * *P* < 0.05, ** *P* < 0.01, and *** *P* < 0.01 compared to TGF-β1-stimulated cells.

### 3.9 Effect of Uvaol, SiO_2_/EuTTA/ZIF-8, and SiO_2_/EuTTA/ZIF-8-Uvaol on TGF-β1-Induced Migration in A549 Cells

To determine whether the effects of uvaol and its nanocarrier on cell morphology also influenced functional cellular responses, we evaluated the impact of SiO_2_/EuTTA/ZIF-8-uvaol nanocomposites on the migratory capacity of A549 cells stimulated with TGF-β1. Cell migration is a hallmark of epithelial-mesenchymal transition (EMT) and is closely linked to pathological processes such as tissue remodeling and fibrosis. By examining the influence of the nanocomposite on cell migration, we aimed to validate its potential as a therapeutic agent for mitigating chronic inflammation-associated functional changes.

Scratch assays were conducted to evaluate the effects of uvaol and SiO_2_@EuTTA@ZIF-8-uvaol on TGF-β1-induced cell migration. Confluent A549 cell monolayers were scratched and treated with vehicle (control), uvaol, or SiO_2_@EuTTA@ZIF-8-uvaol at 0.1 μg/mL. As chown in Figure 15A, cell migration into the scratched area was assessed at 0 and 24 h. TGF-β1 stimulation (5 ng/mL) significantly enhanced cell migration, resulting in a wound closure rate of 29% compared to that in vehicle-treated cells (Figure 15B). Neither uvaol alone nor the SiO_2_@EuTTA@ZIF-8 nanocarriers affected TGF-β1-induced migratory response. However, treatment with the SiO_2_@EuTTA@ZIF-8-uvaol nanocomposite notably reduced cell migration to the scratched area, achieving a 32% inhibition rate (Figure 15B). These findings demonstrate that the nanocarrier effectively potentiates the antimigratory effects of uvaol, highlighting its therapeutic potential in limiting cell migration associated with TGF-β1-induced epithelial-mesenchymal transition.

**Figure 15:**
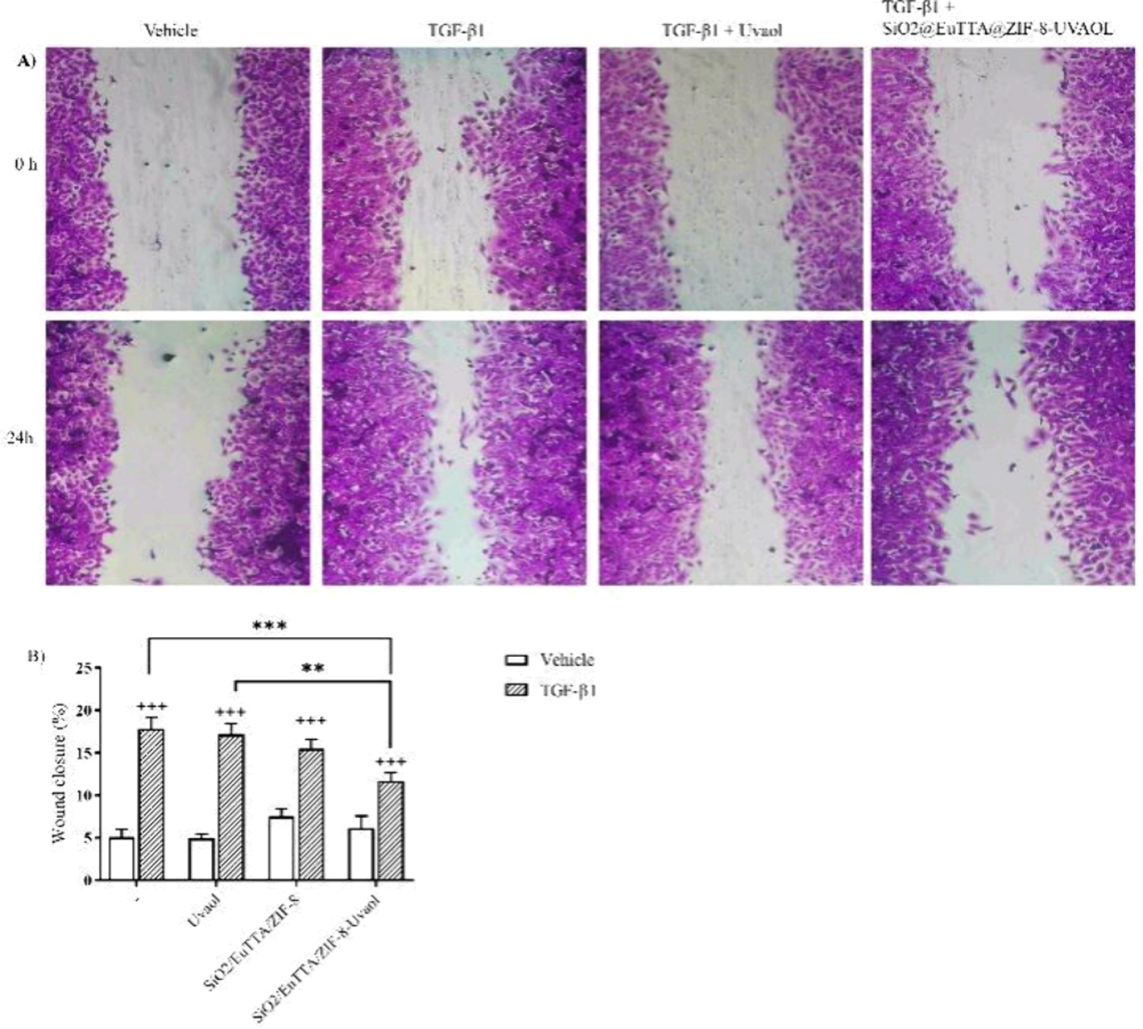
Reduction of TGF-β1-induced cell migration. Cells were treated with (0.1 μg/mL) uvaol, SiO_2_/EuTTA/ZIF-8, and SiO_2_/EuTTA/ZIF-8-uvaol, and images were captured to calculate the scratch closure. Representative photomicrography images showing the scratched area of cells treated with vehicle (control) or uvaol, and cell migration toward the cell-free area after 24 h (Magnification 10×; panel A). Percentage of scratch covered was measured by quantifying the total distance that cells moved from the edge of the scratch toward the center of the scratch, using ImageJ software, followed by conversion to a percentage of the wound that was covered (panel B). Bars represent means ± standard error of the mean (S.E.M.) of three independent experiments. Statistical significance among groups was determined by one-way ANOVA followed by Tukey’s post-test. +++*P* < 0.001 compared to respective vehicle-treated cells; ** *P* < 0.01 and *** *P* < 0.001.

## 4 Conclusion

This study demonstrates that the SiO_2_/EuTTA/ZIF-8 system is a promising candidate for controlled drug delivery. Optical characterization confirmed that the photoluminescence properties of EuTTA were preserved within the ZIF-8 framework. Adsorption studies revealed efficient uvaol loading with a high capacity observed at elevated drug concentrations. The release profile indicated a controlled drug release, highlighting its suitability for sustained delivery in physiological environments. Cytotoxicity assays showed that the SiO_2_/EuTTA/ZIF-8 system was nontoxic at concentrations below 10 μg/mL but exhibited toxicity at higher levels. Molecular docking simulations suggested that uvaol interacted with the surface of ZIF-8 through hydrophobic and π–π stacking interactions, contributing to the stability of the system. In conclusion, the SiO_2_/EuTTA/ZIF-8 system demonstrated great potential for drug delivery applications, combining favorable release properties, biocompatibility, and stability, making it particularly suitable for phototherapy and combination therapies.

## Supplementary Material



## Notes

UV–vis absorption spectrum and calibration curve of uvaol; SEM and DLS particle size histograms for SiO_2_, ZIF-8, SiO_2_/EuTTA, and SiO_2_/EuTTA/ZIF-8; proposed reaction mechanism for SiO_2_ synthesis; excitation and emission spectra, exponential decay curves for EuTTA complex; cumulative UVAOL release curves fitted to kinetic models (PDF)

## Abbreviations

CCR2: CC chemokine receptor 2
CCL2: CC chemokine ligand 2
CCR5: CC chemokine receptor 5
TLC: thin layer chromatography
