# Supplementary material for: Luminescent Nanocomposite SiO2/EuTTA/ZIF‑8 Loaded with Uvaol: Synthesis, Characterization, Anti-Inflammatory Effects, and Molecular Docking Analysis
Source: ACS Omega. 2025 Jul 14;10(29):32391–403. doi: 10.1021/acsomega.5c04682 (PMC12311653; doi:10.1021/acsomega.5c04682)
Supplement: Supplementary file 1 [file ao5c04682_si_001.pdf]

# Luminescent Nanocomposite $SiO_2$/EuTTA/ZIF-8 Loaded with Uvaol: Synthesis, Characterization, Anti-Inflammatory Effects, and Molecular Docking Analysis

Rodrigo S. Viana[1,2*], Polliane Maria Cavalcante de Araújo[3], Juliane Pereira da Silva[3], Jordana Rodrigues de Santana[3], Erick Gabriel Alves Ferreira[3], Alef Batista Bezerra Barros[3], Cintya D' Angeles do Espírito Santo Barbosa[4], Carlos Américo Lechuga Puma[5], Larissa T. Jesus[6], Ricardo O. Freire[6], J. Michael Mathis[7], Emiliano Barreto[3]**, and Severino Alves Junior[2]

[1]Technology Center, Federal University of Alagoas, Campus A.C. Simões, Tabuleiro dos Martins, Maceió, Alagoas, 57072-900, Brazil.

[2]Department of Fundamental Chemistry, Federal University of Pernambuco, 50670-901, Recife, Pernambuco, Brazil.

[3]Laboratory of Cell Biology, Federal University of Alagoas, 57072-900, Maceió, Alagoas, Brazil.

[4]Institute of Chemistry and Biotechnology, Federal University of Alagoas, A.C. Simões Campus, Tabuleiro dos Martins, Maceió, Alagoas, 57072-970, Brazil.

[5]Pharmacy Department, Federal University of Alagoas, Campus A.C. Simões, Tabuleiro dos Martins, Maceió, Alagoas, 57072-900, Brazil.

[6]Pople Computational Chemistry Laboratory, Department of Chemistry, Federal University of Sergipe –UFS, 49100-000 São Cristóvão, SE, Brazil.

[7]School of Biomedical Sciences, University of North Texas Health Science Center, Fort Worth, TX 76107, USA.

AUTHOR ADDRESS: *rs.vianarodrigo@gmail; **emilianobarreto@icbs.ufal.br.

# Supplementary Information

## Figure S1

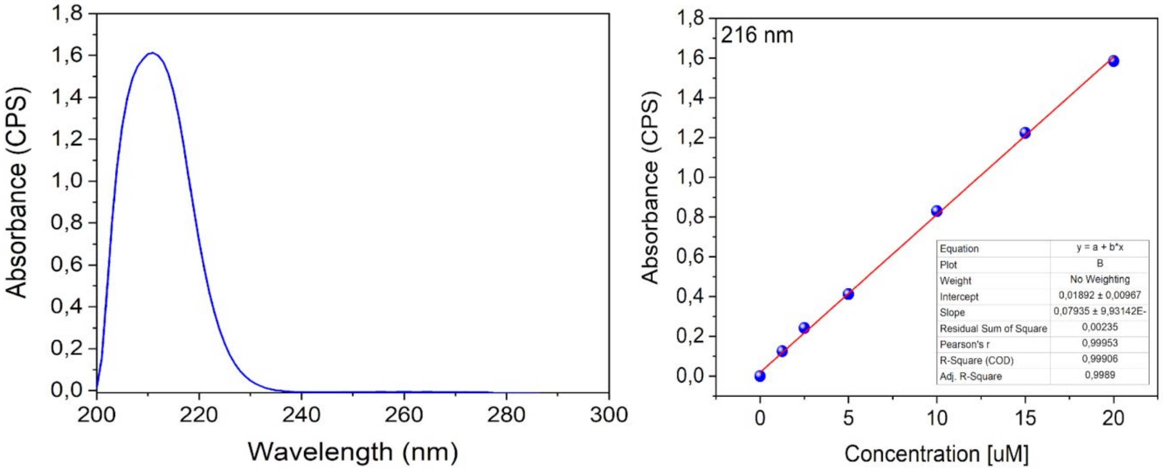


**Figure S1:** UV-Vis absorption spectrum of UVAOL in 20 μM PBS (left) and the calibration curve of UVAOL within the concentration range of 0-20 μM (PBS; pH 7.4) (right).

**Figure S2**

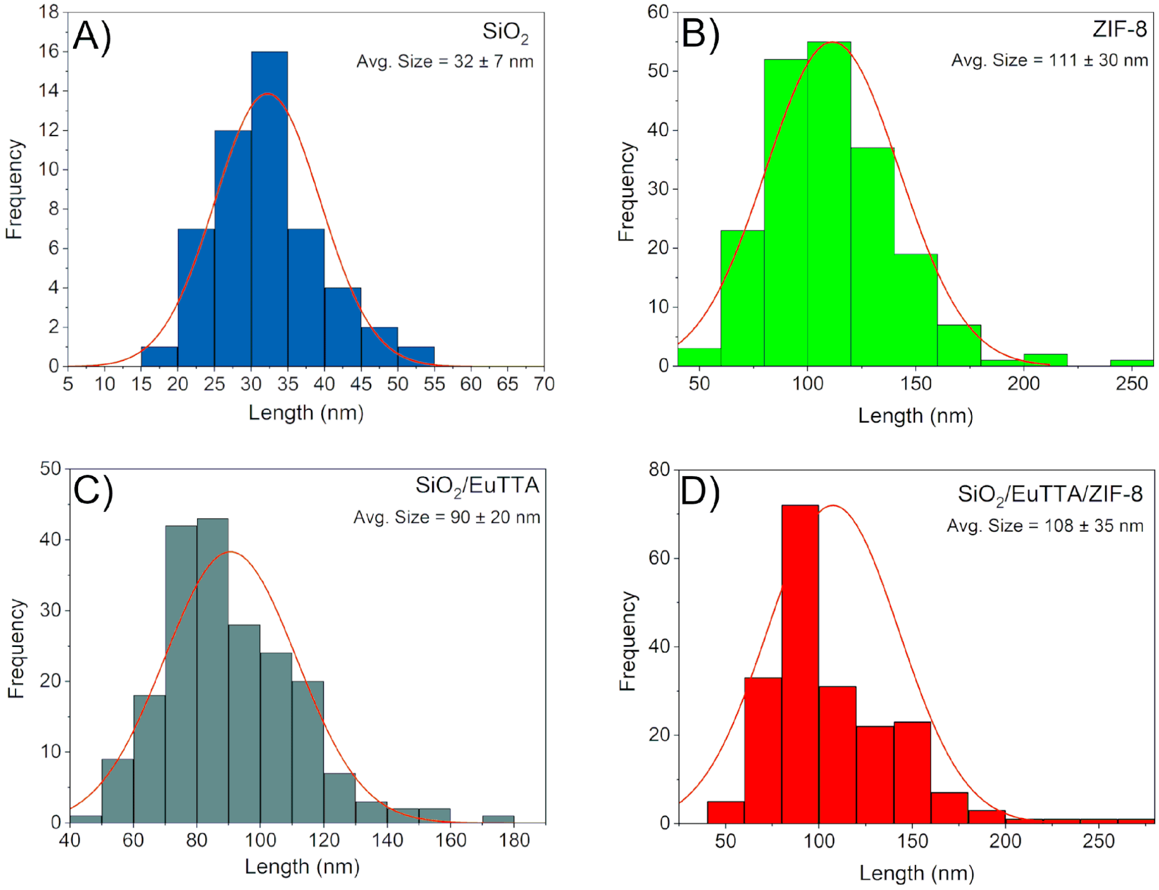


**Figure S2:** Histogram distribution of particle size obtained from SEM microscopy for $SiO_2$ nanoparticles, ZIF-8, $SiO_2$/EuTTA, and $SiO_2$/EuTTA/ZIF-8 samples.

## Figure S3

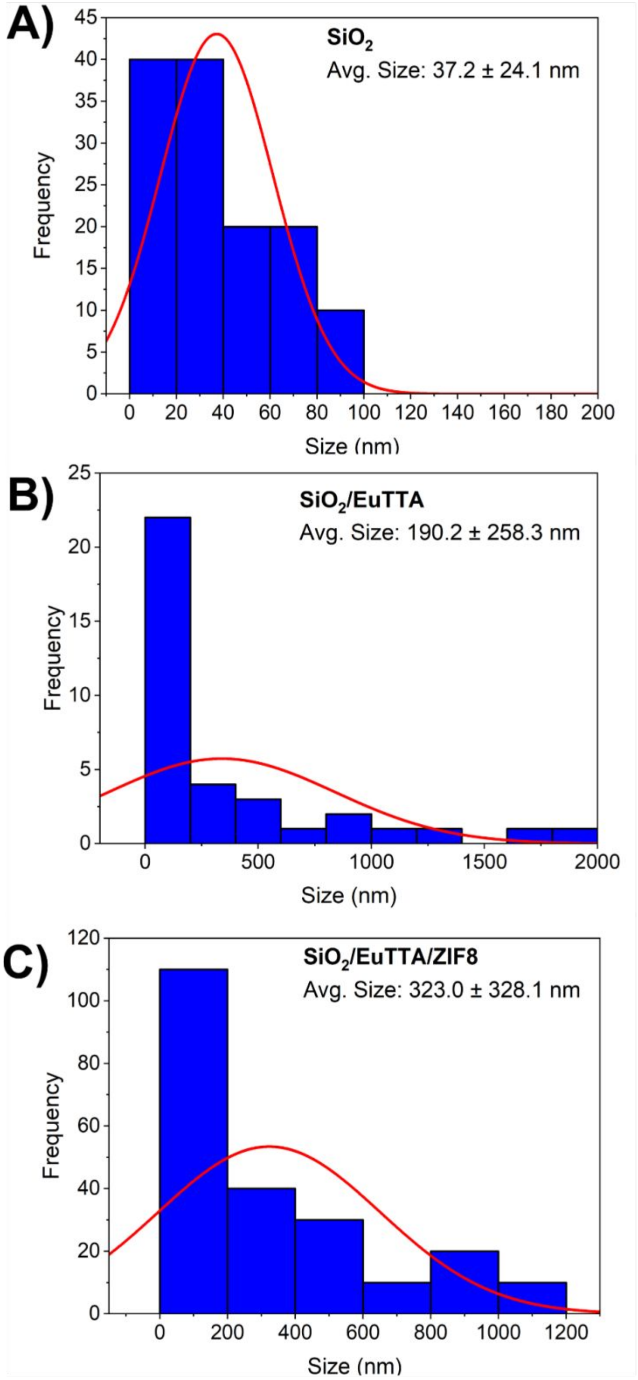


**Figure S3:** Histogram of particle size distribution from Dynamic Light Scattering (DLS) for $SiO_2$, $SiO_2$/EuTTA, and $SiO_2$/EuTTA/ZIF-8.

## Figure S4

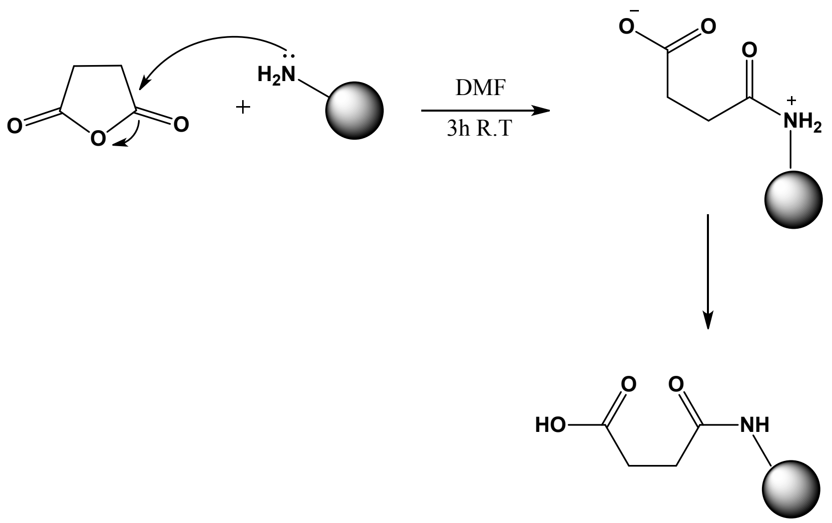


**Figure S4:** Proposed reaction mechanism for the synthesis of $SiO_2$ nanoparticles.

## Figure S5

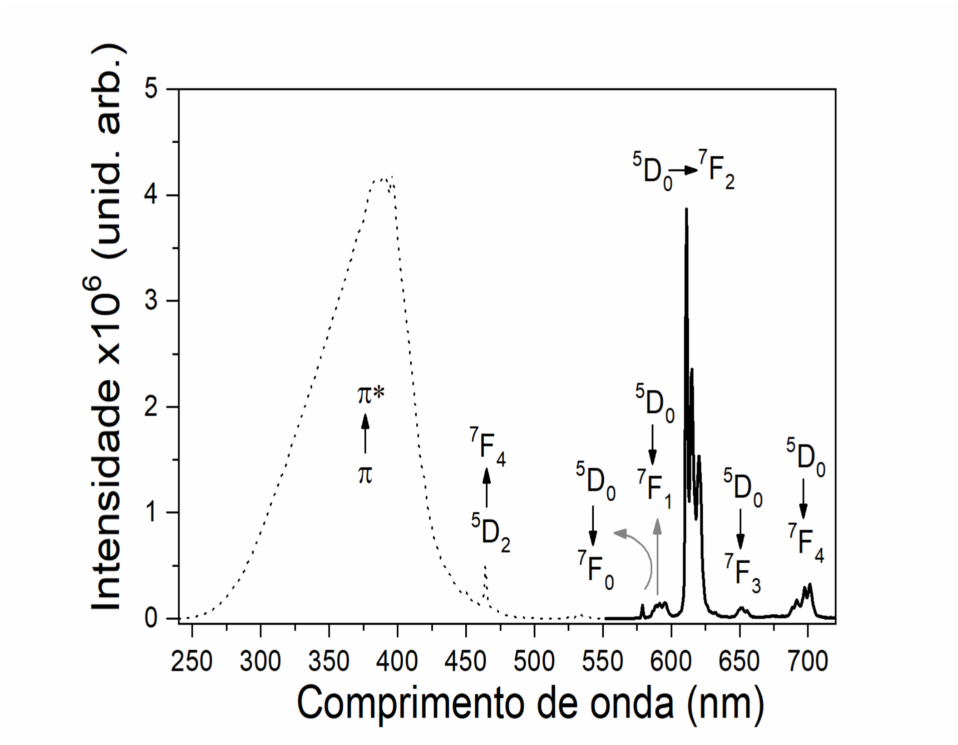


**Figure S5:** Excitation ($\lambda_{Em}$ = 611 nm; dotted line) and emission ($\lambda_{Ex}$ = 397 nm; full line) spectra for EuTTA complex.

## Figure S6

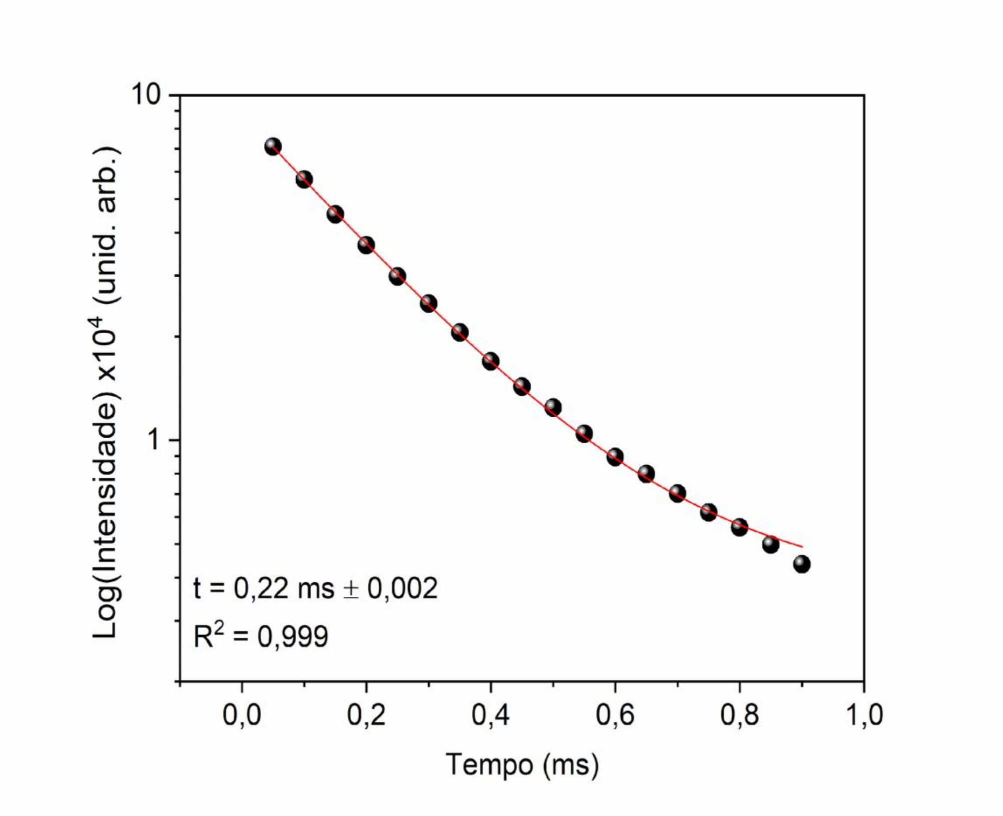


**Figure S6:** Exponential decay curves ($\lambda_{Ex}$ = 397 nm and $\lambda_{Em}$ = 611 nm) for EuTTA recorded at room temperature.

**Figure S7**

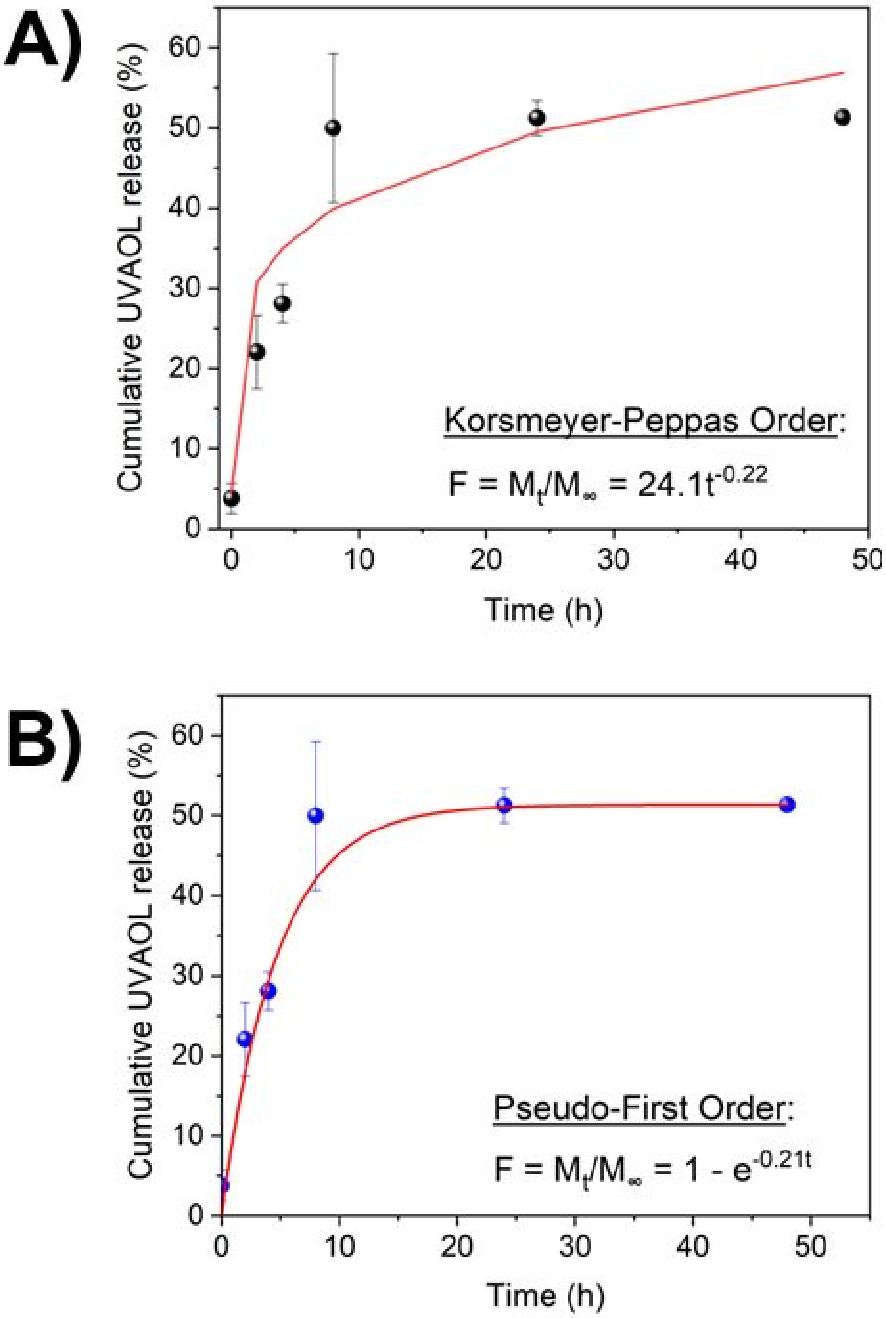


**Figure S7:** Cumulative UVAOL release curves of UVAOL from the $SiO_2$/EuTTA/ZIF-8 nanocomposite fitted to the A) Korsmeyer-Peppas model and the B) Pseudo-First Order model
